# Analyzing the queuing theory at the emergency department at King Hussein cancer center

**DOI:** 10.1186/s12873-023-00778-x

**Published:** 2023-03-01

**Authors:** Mahmoud Salameh Qandeel, Islam Khaleel Al-Qudah, Riyad Nayfeh, Haitham Aryan, Omar Ajaj, Hisham Alkhatib, Yousef Hamdan

**Affiliations:** 1 Department of Health Informatics, King Hussein Cancer Center, Amman, Jordan; 2 Doctoral School of Business Administration, Faculty of Business and Economics, University of Pécs, Pécs, Hungary; 3 Department of Nursing, King Hussein Cancer Center, Amman, Jordan

**Keywords:** KHCC, ED, Health informatics desk, Triage room, Emergency bed area

## Abstract

**Objectives:**

This study was conducted in 2022 at King Hussein Cancer Center (KHCC) to analyze the queuing theory approach at the Emergency Department (ED) to estimate patients’ wait times and predict the accuracy of the queuing theory approach.

**Methods:**

According to the statistics, the peak months were July and August, with peak hours from 10 a.m. until 6 p.m. The study sample was a week in July 2022, during the peak days and hours. This study measured patients’ wait times at these three stations: the health informatics desk, triage room, and emergency bed area.

**Results:**

The average number of patients in line at the health informatics desk was not more than 3, and the waiting time was between 1 and 4 min. Since patients were receiving the service immediately in the triage room, there was no waiting time or line because the nurse’s role ended after taking the vital signs and rating the patient’s disease acuity. Using equations of queuing theory and other relativistic equations in the emergency bed area gave different results. The queuing theory approach showed that the average residence time in the system was between 4 and 10 min.

**Conclusions:**

Conversely, relativistic equations (ratios of served patients and departed patients and other related variables) demonstrated that the average residence time was between 21 and 36 min.

**Supplementary Information:**

The online version contains supplementary material available at 10.1186/s12873-023-00778-x.

## Background

The healthcare sector is unlike other organizations because the connections between input and output are varied and not strictly based on time. For clarification, the bottleneck is noticeable in some areas of hospitals because of the variety of cases and the required time to serve patients, such as waiting for an appointment in a radiology department, the ED, or other clinical sectors. Researchers, therefore, need some improvements to reduce waiting times and take better care of patients. Many issues could prevent a hospital from being efficient: the variability of patients' arrival times; giving special priority to some cases, such as life-threatening ones, at the expense of others; and the utilization of the hospital's systems [1]. The ED is considered a vitally productive unit. In addition to concerns about patient satisfaction and patients left without being seen, waiting too long poses a risk to the patient's health. Therefore, this malfunction of the system's capacity or processes affects the patients' state of mind and health [2]. As a result, researchers discovered that patients who stay in the ED for an extended period before being treated or admitted request to be discharged against medical advice. In some cases, the mortality rate rises [3, 4]. Undoubtedly, one of the main routes to a hospital is the ED. Unplanned patient arrivals are highly unpredictable and not even under control. Alternatively, arriving during peak hours can cause a bottleneck that endangers patients' lives or health. Consequently, this pressure on staff and inadequate types of equipment lead to more mistakes, and patients could wait a long time. Because of this, staff members occasionally resort to "fast-track treatment". On the other hand, patients who have to wait for a long time may be exposed to more infectious diseases, while their health could worsen for those who leave the ED without receiving the treatment [5]. To uncover the status quo, in some areas at work, the waiting times and queue lines indicate that the demand for a service outweighs the current capacity. As a result, queuing theory helps reveal the problem and intervene to increase the capability to reflect the magnitude of demand and prevent an area of work from being inefficient [6]. In a case where patients in emergency departments (EDs) in the USA had to wait slightly less than an hour for treatment, approximately 2% of patients left the hospital without treatment due to the long waiting list, and more than 2 million visits were lost [2]. Significantly, patients spent approximately 39 min on the day shift and 35 min at night filling medications. Adding one employee reduced the average queue length to approximately ten patients and 18 min of waiting time. The conclusion was to implement a multitask employee and increase the number of staff in the process [7]. In contrast, in Nigeria, in outpatient clinics, patients did not attend on time and ended up not receiving treatment. The suggestion for a suitable queuing system model could help improve patient satisfaction, reduce waiting time, and increase efficiency in healthcare facilities [8]. Similarly, Chen and colleagues [9] applied an algorithmic mobile application and a queuing system, an Apache Spark-based cloud, to a substantial database of patients from many hospitals to foresee the needed time at each point in the treatment process. The experimental results confirmed the application's effectiveness and the reduced waiting time. In a study in a tertiary hospital in Australia, patients with low acuity stayed in the waiting room, and high-acuity patients had priority in the no-waiting area. The study proposed machine learning algorithms and the mean squared to predict the waiting time [10]. However, in another study, the autocorrelation coefficient and Pearson's correlation were used to forecast the crowding of patients in an emergency department. The results showed that, on average, the waiting time was approximately 13 min, the occupancy was 83%, and the length of stay was 6.4 h [11]. Remarkably, however, the need to visit the ED is related to specific seasons or days of the year [12]. Accordingly, Sun and colleagues [13] disclosed that waiting times differ according to the days or weeks. They also recorded the times and dates of patients' treatments and categorized them into three categories, from the most critical to the least. Based on quantile regression and the absolute prediction error, the analysis revealed that strata (1, 2, and 3) composed 6.8%, 41.9%, and 51.3% of the total, respectively. However, after controlling for confounders, the median absolute prediction error for stratum 1 was insignificant for prediction waiting time because the queue size increased. As a result, the shorter the wait time, the faster the flow of patients, with categories 2 and 3 taking 9.2 and 12.9 min, respectively. Otherwise, a study demonstrated that queuing theory predicts admissions and discharge ratios with a correlation coefficient of 0.89. In addition, per month, the demand was + 0.4% to -2.3%, representing the variation between the predicted and observed values. Even though patients arrive at hospitals randomly, queuing theory provides a reliable estimation method [14]. Additionally, in analyzing the queuing theory in an Iranian ED, the aim was to minimize waiting time, so the suggestions were to increase the bed capacity and other required resources, as well as classify patients in terms of disease intensity, which would be better than the medical specialty [15]. Nevertheless, Wiler and colleagues [16] used a pattern depending on queuing theory basics. Measuring the flow of patients by using chi-square and ttests depending on the queuing theory's derivation and validation, this queuing theory proved to predict the effects of patient arrivals, completion rates, and treatment times. Significantly, a study at the British Columbia Cancer Agency of ambulatory care used a simulation of resource distribution, appointment schedules, and operational factors. Depending on the queuing theory analysis for each scenario, the modifications were adopted concurrently, showing that patients' waiting times were reduced by approximately 70% and the required space for the same patients by 25%. The recommendations were to start on time, arrange better patient scheduling, and offer flexible screening areas [17]. However, the researchers had noticed in the ED at the KHCC that patients wait a while before receiving treatment. Thus, there was a need to conduct an analytical study at the ED to help determine the magnitude of waiting times and give recommendations for providing optimal patient care based on the study's results. The purpose of this study, once again, is to determine the statistical waiting time and queuing lines at the ED. In return, the study results would allow the decision-makers at the KHCC to increase the staff, tools, or space. Notably, this study took the accuracy of the waiting theory into account.

## Methods

### Study design

The first step in the process is that patients who come to the ED should register with the health informatics employee, except in urgent cases, when they come by ambulance through a specified gate. At the emergency department, there is the health informatics desk. The employee is on the front line and does lots of work simultaneously, such as registering for coming patients, doing admission procedures, coordinating with nurse staff, checking the insurance or payments before the registration, entering invoices, closing the final visits, giving reports, and answering inquiries. The second spot is the triage room. After registration, patients are taken to the triage room to be estimated and prioritized based on their illness acuity (low, medium, and high). Third, patients sit for a while in the waiting area to get into a vacant bed in the emergency bed area. Sometimes, the bottleneck is noticeable at the health informatics employee desk or the waiting zone for emergency beds. Generally, the ED has two physicians, but from 12 p.m. to 7 p.m. during the week, except Fridays, they become three. The available beds are 20: 1 is for CPR, and 1 is an isolation bed. There is one health informatics employee per shift, and 11 nurses and one of them is for triage. The average number of visits per 24 hours is 100, and approximately 60% are during peak hours.

At the health informatics desk, patients or their families come first to register or sign papers for floor admission. Others ask about issues unrelated to the emergency service, such as the place of radiology or radiotherapy, appointments, admission office, test results, applications, reports, and insurance office. From 10 a.m. to 6 p.m. on Sunday, there was typically just one patient in line at the health informatics desk, and the average wait time was one minute. On Thursday, however, there were three patients and a four-minute wait. Significantly, each patient comes from the health informatics desk, directly heads to, and enters, the triage room. Undoubtedly, in the triage room, the nurse measures patients' vital signs and gives them a rating according to their acuity level (low, medium, or high). Therefore, the nurse's role at triage ends after that, and no action is needed other than waiting for vacant beds in the emergency bed area. Because of this, there were no waiting periods during the operation. Seventy-five percent of patients had low visual acuity, whereas only 3% had high acuity levels. After measuring their vital signs, patients waited in line for a vacant bed in the emergency bed area, except for Saturday, with approximately between 3 and 9 minutes and 2 and 9 patients per hour waiting for the service.

### Data collection

The given statistics showed that at KHCC, the peak hours during the previous three years (2019 to 2021) were from 10 a.m. to 6 p.m.; except on Fridays, there was no overload. Thus, the researchers collected data by counting patients who came to the ED during peak hours and days from 3 areas (the health informatics desk, the triage room, and the emergency bed area). According to studies [7, 18], the authors used queuing theory equations, as shown in Table 1, by adding them to the Excel program and automatically calculating arrival and departure for each defined hour and day. The authors confirmed the accuracy of the given numbers by entering them into the Supositorio.com program [19].

**Table 1:** Queuing theory equations

Code	Definition	Equation
Ρ	Operation rate	$\uprho = \lambda /u$
Wq	The average waiting time in a queue	$\mathrm{Wq}=\uprho /\upmu -\uplambda$
Ws	The whole average time in the ED	$\mathrm{Ws}= 1/\upmu -\uplambda$
Lq	The average number of patients in a queue	$\mathrm{Lq}=\mathrm{ \rho \lambda }/\upmu -\uplambda$
Ls	The average number of patients at a specific point	$\mathrm{Ls}=\uplambda /\upmu -\uplambda$

*Source*: [7, 18]

## Results

### Description of the sample

**Fig. 1:**
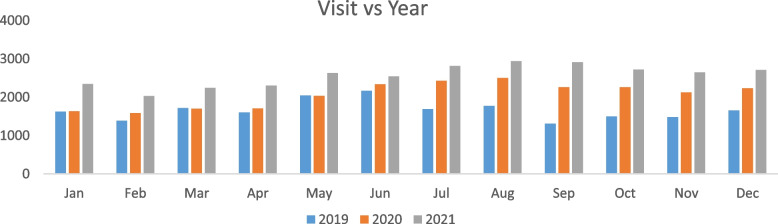
Patient visits from 2019 until 2021

As shown in Table 2 and Fig. 1, the authors collected data from the records for 2019, 2020, and 2021. Indeed, the number of patients has been growing dramatically, from 21,947 in 2019 to 32,841 in 2021. Again, the average number of visits during these years was 1661, 2065, and 2568, respectively. In 2019, May and June were the most crowded months, with 2045 and 2164 visits, respectively. In 2020, the minimum number of visits was in February, with 1582 visits, whereas the maximum was in August, with 2498 visits. Unlike in 2021, visit numbers surged to reach their maximum in August and September, with 2936 and 2911 visits, respectively. Importantly, calculating the average number of visits in the given years per month, the peak influx of patients was in August with 2403 visits, and the least was in February with 1667 visits.

**Table 2:** Patient visits from 2019 until 2021

Month	**2019**	**2020**	**2021**	**Average**
Jan	1620	1633	2345	1866
Feb	1388	1582	2031	1667
Mar	1718	1700	2244	1887
Apr	1599	1705	2299	1868
May	2045	2036	2629	2237
Jun	2164	2337	2539	2347
Jul	1686	2424	2812	2307
Aug	1775	2498	2936	2403
Sep	1307	2255	2911	2158
Oct	1495	2256	2721	2157
Nov	1479	2121	2645	2082
Dec	1652	2232	2708	2197
Total	21,947	26,799	32,841	81,587
Average	1660.667	2064.917	2568.333	2097.97222

*Source*: (The KHCC statistics)

**Fig. 2:**
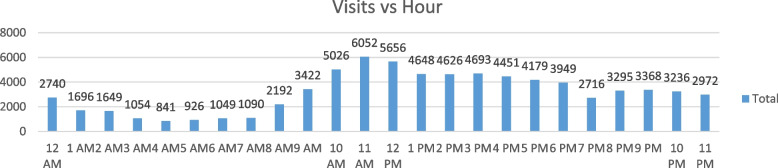
Aggregation visits per hour during the past three years

However, as shown in Fig. 2, calculating the aggregation visits per hour during the past three years gave the authors clear information about the peak hours. The number of visitors gradually increased starting at 9 a.m., with the most pressure on the facility from 10 a.m. to 6 p.m. Additionally, the highest point was at 11 a.m. with 6052 visits, and after that, it dropped twice, at noon with 5656 and at 1 p.m. with 4648, to start leveling out until 6 p.m. with 3949 visits.

**Fig. 3:**
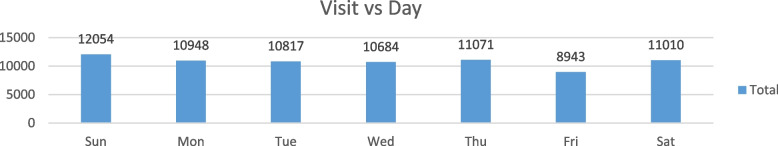
Aggregation visits per day during the past three years

In Fig. 3, looking at the aggregation of visits per day during the past three years, it seems that the highest ratio of visits to the hospital was on Sundays, with 12,054 visits; Fridays were the lowest, with 8943 visits, while on other days of the week, with an insignificant difference, the ratio was between 10,684 and 11,071 visits. Drawing on the given numbers, the authors collected data during a chosen week of July, except for Fridays, which were not significant from 10 a.m. until 6 p.m. during peak hours.

### Analysis of queuing theory and other relativistic equations

#### Health informatics desk

Table 3 shows that the average number of patients who arrived at the health informatics desk during the peak hours, from 10 a.m. to 6 p.m., was a minimum of 51 on Thursday and 73 on Wednesday, where the employee at this point served 111 patients on Sunday compared to the number of patients arriving, which was 63. However, the average number of patients in line was approximately one, with an operating ratio of 57% on Sunday. On Thursday, there were approximately three patients with an operating ratio of 80%. The residence time between arrival and service provision reached a maximum of 5 min.

**Table 3:** Health informatics desk

Day and date	**Arrival time ʎ**	**service time µ**	**Average operation**	**The average number of patients in the line (average queue length)**	**The average number of patients in the system**	**The average wait time in the line**	**Average time in the system (average residence time in the system)**
Saturday 16–7-2022	67	94	0.712766	1.768715524	2.48148148	1.58392435	2.222222222
Sunday 17–7-2022	63	111	0.5675676	0.744932432	1.3125	0.70945946	1.25
Monday 18–7-2022	60	83	0.7228916	1.885804086	2.60869565	1.88580409	2.608695652
Tuesday 19–7-2022	62	85	0.7294118	1.966240409	2.69565217	1.9028133	2.608695652
Wednesday 20–7-2022	73	93	0.7849462	2.865053763	3.65	2.35483871	3
Thursday 21–7-2022	51	63	0.8095238	3.44047619	4.25	4.04761905	5

#### Triage room

Importantly, Table 4 shows the number of patients who came to the triage room after registering at the health informatics desk during peak hours; the maximum was on Wednesday (68 patients). The lowest was on Thursday (50 patients). The operating ratio during the week of the study was 100% without any numbers or waiting times. Approximately 75% of patients had a lower level of illness, 21% had a medium case, and 4% had a high acuity level.

**Table 4:** Triage room

Day and Date	**Arrival time ʎ**	**service time µ real**	**Average operation**	**The average number of patients in the line (average queue length)**	**The average number of patients in the system**	**The average waiting time in the line**	**Average time in the system (average residence time in the system)**	**Low disease intensity, according to nurse evaluation**	**Medium disease intensity, according to nurse evaluation**	**High disease intensity, according to nurse evaluation**	**Total patients**
Saturday 16–7-2022	54	54	1	0	0	0	0	22	30	2	54
Sunday 17–7-2022	57	57	1	0	0	0	0	45	11	1	57
Monday 18–7-2022	58	58	1	0	0	0	0	40	15	3	58
Tuesday 19–7-2022	58	58	1	0	0	0	0	56	1	1	58
Wednesday 20–7-2022	68	68	1	0	0	0	0	61	4	3	68
Thursday 21–7-2022	50	50	1	0	0	0	0	36	11	3	50

#### Emergency bed area

As shown in Table 5, the average operation was 56% on Saturday, with a minute wait time in line, and approximately 90% on Monday, Tuesday, and Wednesday. From Sunday to Thursday, the average time in the system was between 4 and 10 min. Approximately 3 to 9 patients per hour waited in line for service.

**Table 5:** Applying queuing theory to the emergency bed area

Day and Date	**Arrival time ʎ at the emergency bed**	**Service time µ real**	**Average operation**	**The average number of patients in the line (average queue length)**	**The average number of patients in the system**	**The average wait time in the line**	**The average time in the system (average residence time in the system)**
Saturday 16–7-2022	40	72	0.555555556	0.694444444	1.25	1.041666667	1.875
Sunday 17–7-2022	48	64	0.75	2.25	3	2.8125	3.75
Monday 18–7-2022	57	63	0.904762	8.595238	9.5	9.047619	10
Tuesday 19–7-2022	53	59	0.898305	7.935028	8.833333	8.983051	10
Wednesday 20–7-2022	61	69	0.884058	6.740942	7.625	6.630435	7.5
Thursday 21–7-2022	46	60	0.766667	2.519048	3.285714	3.285714	4.285714286

#### Relativistic analysis of the emergency bed area

In Table 6, the average patient residence was 21 to 36 min. In addition, the average number of patients receiving the service was approximately 2 to 3 per hour. According to the physicians' assessment, the low acuity ratio was 61%, the medium acuity ratio was 25%, and the high acuity ratio was 14%. The patient-physician ratio was between 2 and 3 patients per physician, and the patient-nurse ratio was less than one patient per hour.

**Table 6:** Applying relativistic equations to the emergency bed area

Day and date	**Low disease intensity, according to physician evaluation**	**Medium disease intensity, according to physician evaluation**	**High acuity, according to physician evaluation**	**Total of patients who received the service**	**Patients departure**	**The average patients’ residency per hour (aggregation of the number of patients' arrivals to the beds per hour divided by the departure per hour)**	**The average time for patient residency per hour ((60 min/(aggregation of the number of patients' arrivals to the beds per hour divided by the departure per hour))**	**The physicians treat patients per hour. ((2 physicians available, except (from noon and until 19 o’clock, 3 physicians))**	**The nurse treats patients per hour. (10 nurses) during the day shift**
Saturday 16–7-2022	43	17	12	72	38	1.894736842	31.66666667	3.375	0.9
Sunday 17–7-2022	34	22	8	64	28	2.285714	26.25	1.93125	0.8
Monday 18–7-2022	34	16	13	63	38	1.657895	36.19048	3.020833	0.7875
Tuesday 19–7-2022	33	17	9	59	33	1.787879	33.55932	2.75	0.7375
Wednesday 20–7-2022	51	11	7	69	28	2.464286	24.34783	3.25	0.8625
Thursday 21–7-2022	41	14	5	60	21	2.857143	21	2.854167	0.75

*Source*: (authors’ elaboration)

## Discussion

As mentioned early in the data analysis, with 2045 and 2164 visits, May and June 2019 had the most visitors. In 2020, February saw the fewest visits, with only 1582, while August saw the most, with 2498. Contrary to 2021, the number of visits increased dramatically, peaking at 2936 and 2911 in August and September, respectively. Typically, based on the average number of visits made in the three given years per month, August saw the most considerable influx of patients with 2403 visits, and February saw the lowest with 1667 visits. There have been no published studies on queuing theories applied at the ED at the KHCC, but the management is always prepared to provide enough staff and use more space to accommodate the changing demand. Gradually increasing, there were two doctors on the day shift, now three during peak hours, and now the nursing staff is eleven. In some cases, when patients arrive at the same time unexpectedly, assistance from the inpatient department is requested, and occasionally, an area called the walk-in area is temporarily opened for patients when there is congestion or no vacant beds on the inpatient floors. This study agrees with Sun and colleagues [13] because they confirmed that waiting times differ according to the time of day or week. On Sunday, the average queue length at the health informatics desk was roughly one patient, from 10 a.m. until 6 p.m., and the average waiting time in the line was one minute. On Thursday, there were three patients and four minutes. As seen in the results at triage, the operation was 100% successful, with no waiting times. Approximately 75% of patients had low, and 3% had high acuity. The authors assured task specialization; in contrast, Bahadori and colleagues [7] suggested implementing a multitasking employee. To give a clear picture of the workflow of the ED, the authors use some relativistic equations (ratios of served and departed patients and other related variables). Surprisingly, in this study, the results of the queuing theory according to the average residency time at the maximum were approximately 10 min. Similar to McManus and colleagues [14], we applied the median absolute prediction error; the waiting time for low and medium acuity was between 9 and 13 min. However, again, using relativistic equations showed that it was between 21 and 36 min, and the average number of patients receiving the service was approximately 2 to 3 per hour. Hence, that could represent a factual comparison related to the number of beds, staff, and physicians in the emergency bed area and the fast-track treatment of cases. However, Pak, Gannon, and Staib [10] revealed that the waiting time was between 21 and 28 min. Conversely, Hoot and colleagues [11] showed that the waiting time was approximately 13 min, the occupancy was 83%, and the length of stay was 6.4 h.

## Conclusions and recommendations

The first point of contact in the emergency department is the health informatics desk, where the average waiting time in line during peak hours was between 1 and 4 min. To reduce the waiting time in line, the authors suggest making informative boards with clear instructions at the main doors of the hospital about the structure and vital places that should serve patients, such as the reception to answer patients' queries and the patient affairs office to release patients' information and test results. Second, since every patient could reach the triage room and receive service, the waiting time was zero. Hence, patients should be separated, and those with fast-track treatment should, if possible, take the prescribed medicine at the triage or other room allocated for this purpose to reduce the burden on the emergency bed area. Finally, the average time for patient residency ranged between 21 and 36 min, calculated by dividing the service received by the number of patient departures. Ironically, the mentioned numbers went against the computed numbers of the queuing theory at the emergency room, which were between 4 and 10 min, except for Saturday. As a result of testing the accuracy of queuing theory, this study's results contradict the findings of Mcmanus and colleagues [14] and Wiler and colleagues [16]. Therefore, researchers should conduct more comparative studies between queuing theory and other related methods. However, opening more space (beds, staff) for the emergency bed area will be better if the waiting time is not satisfying for the patients. Additionally, they should conduct a comparative study after that to measure any enhancements. Seriously, an unjustified delay in serving patients due to slow procedures may affect their health, the reputation of the hospital, and the satisfaction of staff and patients [20].

## Supplementary Information

**Additional file 1.**

## Data Availability

All data generated and analyzed during this study are included in it and its supplementary information file.
